# Endoscopic ultrasound-guided hepaticogastrostomy without tract dilation using a novel 0.035-inch guidewire

**DOI:** 10.1055/a-2535-1881

**Published:** 2025-02-20

**Authors:** Ritsuko Oishi, Haruo Miwa, Kazuki Endo, Hiromi Tsuchiya, Yuichi Suzuki, Kazushi Numata, Shin Maeda

**Affiliations:** 1 26437 Gastroenterological Center, Yokohama City University Medical Center, Yokohama, Japan; 2 Department of Gastroenterology, Yokohama City University Graduate School of Medicine, Yokohama, Japan

Biliary peritonitis is one of the complications of endoscopic ultrasound-guided hepaticogastrostomy (EUS-HGS), and it is mostly caused by tract dilation 1 2 3 . In patients with acute cholangitis, bile leakage may cause refractory infection in the abdominal cavity. Therefore, omitting tract dilation is expected to reduce the risk of bile peritonitis; however, plastic stent placement without tract dilation has been reported as challenging 4 . A novel 0.035-inch guidewire (CAPELLA 0.035; Japan Lifeline Co., Ltd., Tokyo, Japan) has a stiff shaft that facilitates stent deployment in EUS-HGS ( Fig. 1 ), which is also compatible with most devices designed for 0.025-inch guidewires. Herein, we present two cases in which a plastic stent was successfully placed without tract dilation using a CAPELLA 0.035 during EUS-HGS ( Video 1 ).

**Fig. 1:**
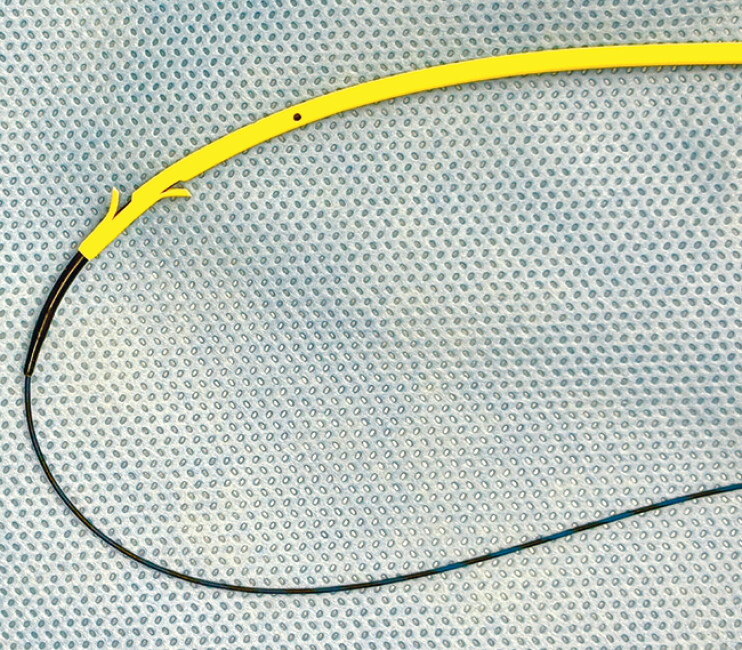
A novel 0.035-inch guidewire (CAPELLA 0.035) has a stiff shaft that facilitates stent deployment in endoscopic ultrasound-guided hepaticogastrostomy, and it is also available with most devices dedicated to 0.025-inch guidewires.

A novel guidewire, CAPELLA 0.035-inch, has a soft and tapered tip that facilitates plastic stent placement without tract dilation during endoscopic ultrasound-guided hepaticogastrostomy.Video 1

Case 1: A 78-year-old man with acute cholangitis caused by biliary stricture of the lateral branch was admitted. EUS-HGS was performed because transpapillary drainage failed. Firstly, B2 was punctured with a 19-gauge needle, and a 0.025-inch guidewire (VisiGlide 2; Olympus Medical Systems, Tokyo, Japan) was inserted after contrast injection. Subsequently, an ultra-tapered catheter (MTW Endoskopie Manufaktur, Wesel, Germany) was advanced, and the guidewire was exchanged for a CAPELLA 0.035. Finally, a 7-Fr plastic stent (Through and Pass Type IT; Gadelius Medical, Tokyo, Japan) was successfully placed ( Fig. 2 ).

**Fig. 2:**
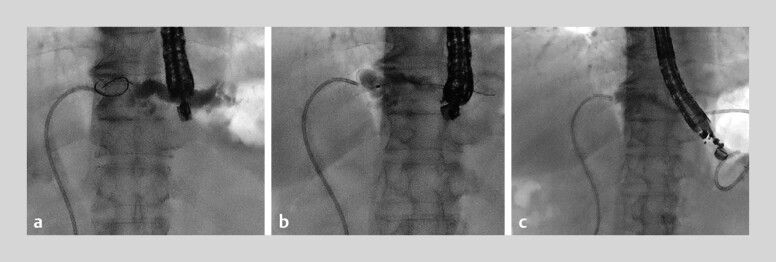
Case 1. **a** B2 was punctured with a 19-gauge needle, followed by contrast injection and insertion of a 0.025-inch guidewire. **b** An ultra-tapered catheter was inserted into the bile duct, and the guidewire was exchanged for a CAPELLA 0.035. **c** A 7-Fr plastic stent was successfully placed without tract dilation.

Case 2: An 82-year-old woman with a hepaticojejunostomy anastomotic stricture due to recurrence of ampullary carcinoma was admitted with acute cholangitis. EUS-HGS was performed for acute cholangitis caused by the recurrence of a biliary obstruction after plastic stent placement. B2 was punctured with a 19-gauge needle and a 0.025-inch guidewire was placed in the right hepatic duct. After the guidewire exchange for a CAPELLA 0.035, the plastic stent was successfully placed ( Fig. 3 ).

**Fig. 3:**
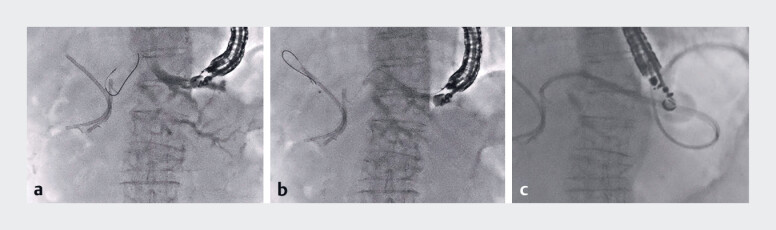
Case 2. **a** B2 was punctured with a 19-gauge needle, followed by contrast injection and insertion of a 0.025-inch guidewire. **b** After exchanging the guidewire for a CAPELLA 0.035, a catheter was inserted into the right hepatic duct. **c** A 7-Fr plastic stent is successfully placed without tract dilation.

To the best of our knowledge, this is the first report of EUS-HGS omitting tract dilation enabled by a novel 0.035-inch guidewire that is essential for this procedure.

Endoscopy_UCTN_Code_TTT_1AS_2AH

## Notes

Endoscopy E-Videos https://eref.thieme.de/e-videos
*E-Videos* is an open access online section of the journal *Endoscopy* , reporting on interesting cases and new techniques in gastroenterological endoscopy. All papers include a high-quality video and are published with a Creative Commons CC-BY license. Endoscopy E-Videos qualify for HINARI discounts and waivers and eligibility is automatically checked during the submission process. We grant 100% waivers to articles whose corresponding authors are based in Group A countries and 50% waivers to those who are based in Group B countries as classified by Research4Life (see: https://www.research4life.org/access/eligibility/ ). This section has its own submission website at https://mc.manuscriptcentral.com/e-videos .
